# China material stocks and flows account for 1978–2018

**DOI:** 10.1038/s41597-021-01075-7

**Published:** 2021-11-25

**Authors:** Lulu Song, Ji Han, Nan Li, Yuanyi Huang, Min Hao, Min Dai, Wei-Qiang Chen

**Affiliations:** 1 Key Lab of Urban Environment and Health, Institute of Urban Environment, Chinese Academy of Sciences, 1799 Jimei Road, Xiamen, Fujian Province 361021 P. R. China; 2 Xiamen Key Lab of Urban Metabolism, Xiamen, Fujian Province 361021 P. R. China; 3 University of Chinese Academy of Sciences, No.19 (A) Yuquan Road, Shijingshan District, Beijing, 100049 P. R. China; 4 Shanghai Key Lab for Urban Ecological Processes and Eco-Restoration, School of Ecological and Environmental Sciences, East China Normal University, 500 Dongchuan Road, Shanghai, 200241 P. R. China; 5 Institute of Eco-Chongming, 3633N. Zhongshan Road, Shanghai, 200062 P. R. China; 6 College of Civil and Transportation Engineering, Shenzhen University, 3688 Nanhai Road, Shenzhen, Guangdong Province 518060 P. R. China; 7 College of Life Sciences, Ningde Normal University, 1 Xueyuan Road, Ningde, Fujian Province 352106 P. R. China; 8 Fudan Tyndall Center, Department of Environmental Science & Engineering, Fudan University, 220 Handan Road, Shanghai, 200438 P. R. China

**Keywords:** Society, Sustainability

## Abstract

As the world’s top material consumer, China has created intense pressure on national or global demand for natural resources. Building an accurate material stocks and flows account of China is a prerequisite for promoting sustainable resource management. However, there is no annually, officially published material stocks and flows data in China. Existing material stocks and flows estimates conducted by scholars exhibit great discrepancies. In this study, we create the Provincial Material Stocks and Flows Database (PMSFD) for China and its 31 provinces. This dataset describes 13 materials’ stocks, demand, and scrap supply in five end-use sectors in each province during 1978–2018. PMSFD is the first version of material stocks and flows inventories in China, and its uniform estimation structure and formatted inventories offer a comprehensive foundation for future accumulation, modification, and enhancement. PMSFD contributes insight into the material metabolism, which is an important database for sustainable development as well as circular economy policy-making in China. This dataset will be updated annually.

## Abstract (summary)

**Table:** 

Measurement(s)	Material stocks • Material flows
Technology Type(s)	digital curation • Mathematical Model
Sample Characteristic - Location	China

Machine-accessible metadata file describing the reported data: 10.6084/m9.figshare.16601249

## Background & Summary

Modern society is enabled by the use of various materials^1,2^. The 20th century is an era of explosion of human civilization and accomplished achievements, which is accompanied by the ever-accelerating annual extraction of biomass by a factor of 3.6, of fossil fuels by 12, of ores and minerals by 27, and of construction materials by 34 times^1,3–6^. Foreseeably, with deepening urbanization in developing countries, the size of material stocks in human societies will continue to grow. According to the reports of the United Nations Environment Program (UNEP), the consumption of biomass, minerals, and ores per year will triple between the present and 2050^7^. With the intensified resource stresses, material metabolism has been the research hotspot of sustainability science^7,8^.

Sustainability science is rapidly evolving and increasingly changing into data-intensive. As one of the concerns of sustainability science, material metabolism, which quantifies the flows of materials and energy into and out of human societies, urgently needs the material stocks and flows data to describe and analyze the metabolic processes^8–10^. Furthermore, based on the material stocks and flows data, material efficiency^11,12^, criticality^13,14^, and recycling^15,16^ may be directly analyzed, and related properties, such as energy consumption^17^, greenhouse gas emissions^18^, and future material demand^19^ can be easily and exactly traced. Hence, an accurate accounting of material stocks and flows data is the premise for achieving the above-mentioned goals.

As the world’s top material consumer, China has created intense pressure on national or global demand for natural resources^7^. With rapid urbanization and industrialization, the materials stocks in China have been experiencing a sharp increasing pattern^4,19–22^, and China’s share of global material stocks increased from 10% to 22%^4^. It has been catching up with developed countries since the 1990s, and only took 20 years to get to the stocks level that industrialized countries had achieved within 70 years. Diverse material stocks have increased exponentially in China: steel stocks increased 2.5-fold in a decade^23^; aluminium stocks rose 100-fold during 1950–2009^24^; copper stocks increased 50-fold during 1952–2015^25,26^; cement stocks increased by 40 times during 1990–2010^21^. China’s accelerating materialization process creates serious environmental problems^27,28^, which makes understanding the material metabolism crucial for implementing effective resource management policies and providing valuable insights into promoting circular economy^9^. However, the material stocks and flows account in China and each province have not been well reported and published. Although previous studies have estimated China’s material stocks^20,21,29–31^, there is considerable disagreement on the stocks level between these studies. For example, Krausmann *et al*.^4^ estimated that the material stocks in China amounted to 136 t/cap in 2010 based upon the top-down approach, while Han and Xiang^19^ estimated the result to be only 33 t/cap in 2008 based upon the bottom-up approach. The difference may be explained by the inconsistent system boundaries, identified end-use sectors, data sources, and estimation approaches, which makes it difficult to clarify the pattern of material use and identify the mechanism of material metabolism.

Considering the great discrepancies of material stocks account and the lack of provincial material stocks and flows account in China, this paper creates the first version of the Provincial Material Stocks and Flow Database (PMSFD), which contains 13 materials’ stocks and flows in five end-use sectors in 31 provinces of mainland China. The spatial scale of PMSFD ranges from province to nation, and the timescale ranges from 1978 to 2018. There are 200,000 + data records stored in the PMSFD, and we also provide the material intensity used in the estimation for transparency and verifiability. By integrating data records into a unified format, PMSFD has taken a step towards overcoming the limited accessibility due to incomplete data availability.

The comprehensive and consistent data records make the PMSTD an important database for sustainability analyses and assessments. For example, PMSTD may be used to facilitate retrospective analyses and prospective forecasts of stocks and flows to assist in identifying and achieving sustainable development. We will advance this goal through this initial version of PMSTD, and update the PMSTD annually. The rest of this paper introduces the estimation approach used to create the PMSFD, the file format used to record the PMSFD, and the properties of the data in the PMSFD.

## Methods

### System boundary

**Fig. 1:**
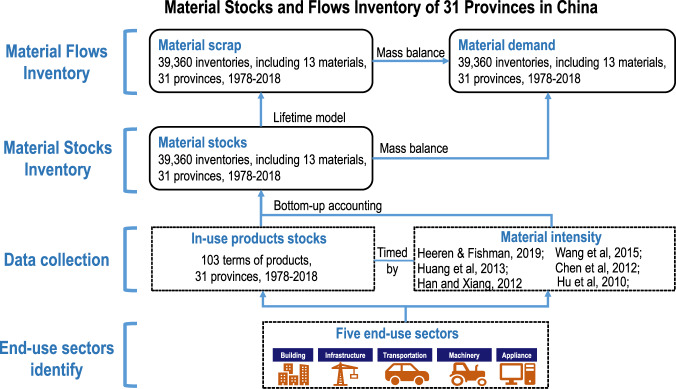
Diagram of material stocks and flows inventory construction.

A PMSF (Provincial Material Stocks and Flows) model, which combines a bottom-up, stocks-drive-flows, and mass-balanced dynamic model, is constructed for material stocks and flows estimates at the provincial level. This combined model can evaluate material inputs, stocks accumulation, and end-of-life outflows. Considering the availability of intensity data and the wide application in society, 13 types of materials, including steel (Fe), aluminium (Al), copper (Cu), rubber, plastic, glass, lime, asphalt, sand, gravel, brick, cement, and wood, are considered in this study. Drawing on a comprehensive provincial product database, annual provincial material uses and flows are estimated. The time horizon is from 1978 to 2018 and all computations are performed in time-discrete steps of one year. The spatial scope covers 31 provinces in mainland China. The workflow is shown in Fig. 1 and is described in detail in the following sections.

### Material stocks

The bottom-up accounting method, which starts from counting every piece of material-containing products, then investigates the material intensity for each product, and finally adds up material contained in all product categories, could complement the top-down method by revealing greater details of technologies and identifying geographical locations of material stocks^23^. Since the physical data of product stocks are becoming available on provincial scale, the bottom-up approach is appropriately adopted to estimate the material stocks ($S\left(t\right)$).

A list of 103 commodities, which are divided into five end-use sectors (including buildings, infrastructure, transportation facilities, machinery, and domestic appliances), is identified and revised based on our earlier study^19^ (Online-only Table 1). Unlike our previous research, we have primarily adjusted the commodities identified in the end-use sectors of buildings and infrastructure. For example, we have added different types of highways, power stations, and cable users in the infrastructure, while removing different structures of residential buildings. The material stocks (${S}_{\bar{C}}$) in each end-use sector ($\bar{C}$) at the time *t* are then calculated as the sum of material stocks of related products (Eq. 1).

1 $${S}_{\bar{C}}\left(t\right)=\sum _{i}{N}_{i}(t)\times {I}_{i}(t)$$

where ${N}_{i}\left(t\right)$ is the amount of product *i* in active use in the end-use sector $\bar{C}$ at time *t* and ${I}_{i}(t)$ is the material intensity of product *i*.

The number of product ${N}_{i}$ quantified by per-household, absolute, or per-capita (Online-only Table 1) is extracted from various official statistical reports, yearbooks, and socio-economic databases (including https://data.stats.gov.cn and http://www.data.ac.cn) for each province during 1979–2019 (see ref. ^19^ for detail). Owing to limited data availability for the individual years, linear interpolation has been used to estimate the missing data between consecutively recorded values^32^.

The material intensity is sourced from various references and estimated based on expert judgements^10,20,23,24,30,33^, which are also provided in our datasets.

The total material stocks $S\left(t\right)$ are calculated as the sum over the five end-use sectors at the time *t* (Eq. 2).

2 $$S\left(t\right)=\sum _{\bar{C}}{S}_{\bar{C}}(t)$$

Residential and non-residential buildings (including buildings of public and industry) are considered when calculating the material stocks in buildings. Unlike residential buildings, the floor space of non-residential buildings is not officially reported in each province. Instead, the floor space of public (${f}_{p}$) and industrial buildings (${f}_{i}$) in towns and townships in each province are recorded by China Urban-Rural Construction Statistical Yearbook for years (http://www.mohurd.gov.cn/xytj/tjzljsxytjgb/jstjnj/index.html)^34^. Hence, we estimate the material stocks in non-residential buildings by assuming that the ratio between non-residential and residential buildings in towns (or townships) applies to the urban (or rural) in each province (Eqs. 3–4).

3 $$R(t)=\left({f}_{p}(t)+{f}_{i}(t)\right)/{f}_{r}(t)$$

4 $${F}_{UR}(t)=\left({A}_{CR}(t)\times P(t)\right)\times R(t)$$

where $R(t)$ is the proportion of floor space of non-residential buildings to residential buildings in urban (or rural) at time *t*, ${f}_{p}(t)$ and ${f}_{i}(t)$ are the floor space of public and industrial buildings in town (or township) for each province at time *t*, ${f}_{r}(t)$ is the floor space of residential buildings in town (or township) for each province at time *t*, ${A}_{CR}(t)$ is the per-capita floor space of residential buildings in (or rural), and $P(t)$ is the urban (or rural) population at time *t*. For the period 1978–2001, ${f}_{p}(t)$, ${f}_{i}(t)$ and ${f}_{r}(t)$ are held constant at the level of 2002, since these data are unavailable before 2002.

In addition, due to limited official reporting data for (large, medium, small, and micro) passenger cars and (heavy, medium, light, and micro) trucks in each province before 2001, we use the proportion of the different sizes of passenger cars and trucks in 2002 to extrapolate the numbers during 1978–2001. Meanwhile, it is difficult to identify the amount of machines used in diverse industries. We evaluate the metal stocks in industrial machinery by supposing that there is a directly proportional relationship between power consumption and the amount of industrial machines, which is recommended by Zhang *et al*.^29^ and Liu *et al*.^32^.

### Material flows

The material outflow at the time *t* is defined as the scrap generated from stocks from the end-use sector $\bar{C}$ of material *m*, and material inflow at the time *t* is defined as inputs to stocks of material *m*. The dynamic stocks-drive-flows model is applied to estimate the material inflows and outflows during 1978–2018 on a sector scale. The annual outflows are determined from stocks using the lifetime model, and the annual inflows are determined from mass balance with outflows and stocks change (Eq. 5):

5 $$demand=inflow=outflow+stock\;change$$

The lifetime distribution expresses the probability of each end-use sector to reach the end-of-life at the time *t*. According to previous studies^15,31^, we assume a normally distributed lifetime $\lambda \left(t,t{\prime} ,L,\sigma \right)$ ($t{\prime}$ = 1949) with end-use sectors dependent mean *L* and standard deviation *σ* (Eq. 6), which determines the outflow ${F}_{out}$ from stocks and inflows ${F}_{in}$ (Eqs. 6–8):

6 $$\lambda \left(t,t{\prime} ,L,\sigma \right)=\frac{1}{\sigma \sqrt{2\pi }}\times exp\left(\frac{-(t-t{\prime} -L)}{2{\sigma }^{2}}\right)$$

7 $${F}_{in}(t)={S}_{\left(t\right)}-{S}_{\left(t-1\right)}+{F}_{out}(t)$$

8 $${F}_{out}({\rm{t}})=\sum _{t{\prime} \le t}{F}_{in}\left(t{\prime} \right)\times \lambda \left(t,t{\prime} ,\tau ,\sigma \right)$$

Since material quality requirements are different between buildings, cars, machines, laptops, and other products, the lifetime can vary greatly. However, it is hard to get a specific lifetime of each product in different regions, we assume that the lifetime of products in the same end-use sector keeps unchanged. The mean lifetime and standard deviation for each end-use sector are given in Table 1. According to Eqs. (6–8), when the net stocks (${S}_{\left(t\right)}-{S}_{\left(t-1\right)}$) less than zero, the ${F}_{in}(t)$ will be negative. Hence, to elaborate the negative data of inflow, we will artificially set ${F}_{in}(t)$ into zero. The ${F}_{out}(t)$ will be equal to the net stocks.

**Table 1:** Mean lifetime and standard deviation for different end-use sectors.

End-use sector	Mean lifetime	Standard deviation
Buildings	50	15.0
Infrastructure	50	15.0
Transportation equipment	25	7.5
Machinery	30	9.0
Domestic appliances	15	4.5

When estimating the inflows and outflows from the year 1978, the stocks and the resulting inflows and outflows before 1978 must be taken into account. These approximations of initial stocks and flows are necessary to make the estimation for the period of 1978–2018 more accurate^4^. Hence, a spin-up period has been implemented, and the length of this spin-up period begins in 1949 when the People’s Republic of China was founded. Due to limited official reporting data that existed in each province before 1978, we use the power function revealed by previous studies^35^ to extrapolate the material stocks during 1949–1977 (Eq. 9).

9 $${S}_{p}\left({t}_{0}\right)={K}_{p1}\times {e}^{\left(K{p}_{2}\times {t}_{0}\right)}$$

where ${S}_{p}\left({t}_{0}\right)$ is the material stocks at the time ${t}_{0}$ in province *p* (during 1949–1977), ${K}_{p1}$ and ${K}_{p2}$ are coefficients of the fitting model in each province.

## Data Records

The PMSFD datasets, which contain five core tables, and each table is provided as xlsx files, are freely available through Figshare^36^ and at the Urban Resources, Ecology & Environment website (UREE, https://uree.org/china-material-stocks-and-flows-account/). The heading of tables in PMSFD is hereafter written in italics. A total of 215,333 data records are contained in the datasets. Of these,

96,864 are the account of products in 31 provinces from 1978 to 2018 [File ‘*Input data*’];

331 are material intensity data for 13 materials from 1978 to 2018 [File ‘*Material intensity*’];

39,360 are national and provincial material stocks inventories (13 materials, from 1978 to 2018) [File ‘*Provincial_mateiral_stocks_1978–2018*’];

39,360 are national and provincial material inflows inventories (13 materials, from 1978 to 2018) [File ‘*Provincial_mateiral_inflows_1978–2018*’];

39,360 are national and provincial material outflows inventories (13 materials, from 1978 to 2018) [File ‘*Provincial_mateiral_outflows_1978–2018*’];

The materials stocks and flows inventories, stored by matrices with 29 columns and 41 rows in each sheet table, have a uniform format. The 29 columns are 13 materials stocks in 5 end-use sectors. The 41 rows represent the 41 years. Table 2 is an example of the material stocks of Beijing, and it includes 13 materials stocks in 5 end-use sectors in 2018.

**Table 2:** Sectoral level of material stocks inventory in Beijing in 2018 (in million tonnes).

	Wood	Cement	Brick	Gravel	Sand	Asphalt	Lime	Glass	Plastic	Rubber	Cu	Fe	Al
**Buildings**	28.9	313.8	224.2	904.0	805.6	2.1	33.8	2.1			0.4	51.3	1.9
**Infrastructure**		17.0			162.0	0.1					0.7	26.4	1.1
**Transport equipment**								0.3	1.4	0.3	0.1	7.0	0.5
**Machinery**											0.007	2.4	0.008
**Domestic appliance**											0.1	0.8	0.07

## Technical Validation

### Data overview

**Fig. 2:**
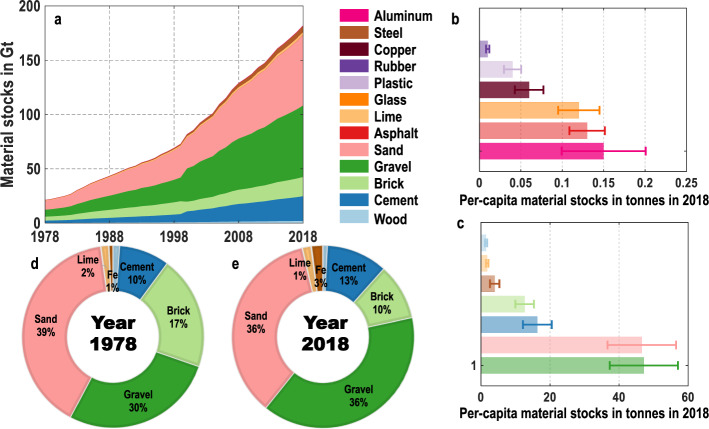
National material stocks during 1978–2018. (**a**) Development of national material stocks by 13 material groups. (**b**), (**c**) Per-capita material stocks in 2018 including uncertainty ranges. (**d**), (**e**) Composition of 13 material stocks in 1978 and 2018. See Tables S1, S2 of the Supplementary Information for total and per-capita material stocks during 1978–2018.

**Fig. 3:**
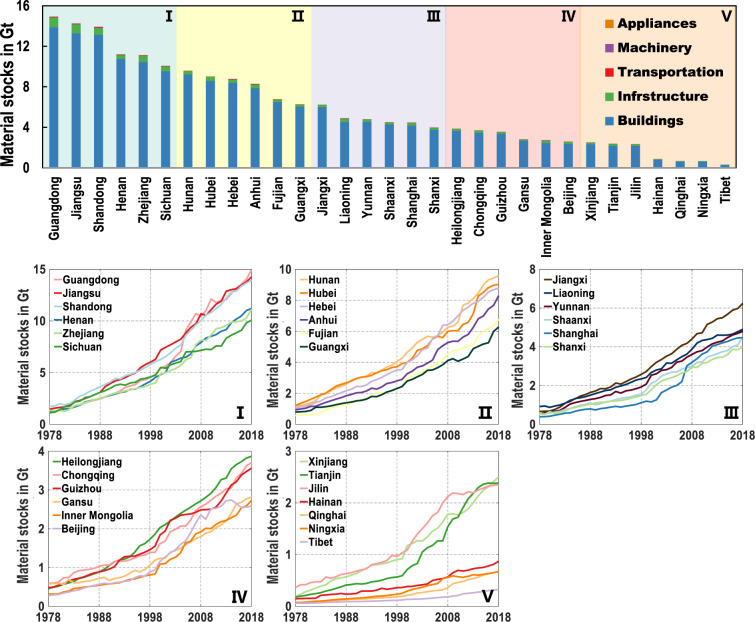
Provincial material stocks during 1978–2018. The upper bar graph is sorted by average stocks in 2018 from the highest to the lowest, which can be divided 31 provinces into five groups from I to V. See Table S3 of the Supplementary Information for provincial material stocks in five end-use sectors. The five lower line charts show the total material stocks of provinces in I to V groups. See Table S4 of the Supplementary Information for provincial material stocks in the past 40 years.

**Fig. 4:**
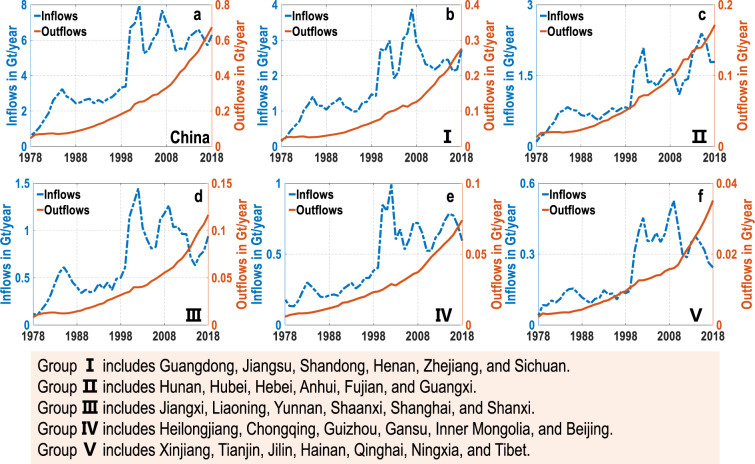
Material demand and scrap dynamics in China (**a**) and five groups (**b**–**f**) during 1978–2018. The five groups from I to V are divided according to stocks level. Refer to Fig. 3 for the details of classification. See Tables S5–S6 of the Supplementary Information for provincial material demand and scrap dynamics in the past 40 years.

**Fig. 5:**
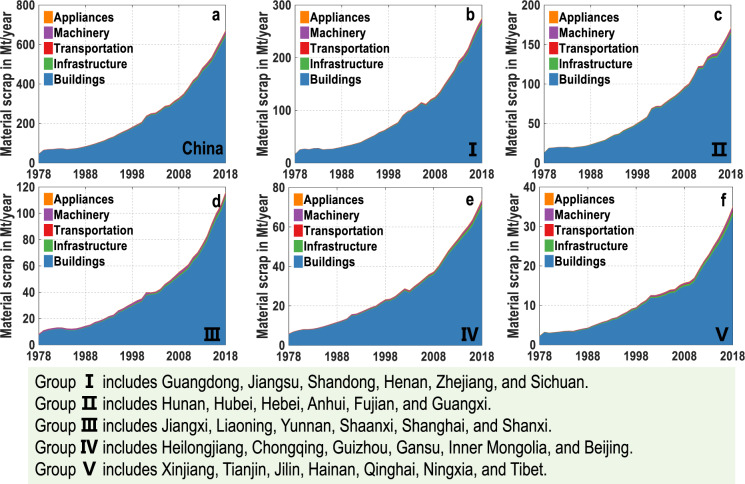
Material scrap supply by sectors in China (**a**) and five groups (**b**–**f**) during 1978–2018. The five groups from I to V are divided according to stocks level. Refer to Fig. 3 for the details of classification. See Table S7 of the Supplementary Information for material scrap clarified by five end-use sectors in the past 40 years.

Nationally, the total material stocks show a continuously increasing pattern especially after 2000, and get to 182.2 Gt in 2018 (Fig. 2a) (Detailed results are available in Table S1 of Supplementary Information). Average per-capita stocks amount to 130.5 t in 2018, and the stocks of gravel are highest (47.2 t/cap) (Fig. 2b,c) (Detailed results are available in Table S2 of Supplementary Information). The composition of material stocks has changed over the past 40 years. The proportion of sand and brick is decreasing, while the proportion of cement and steel is increasing (Fig. 2d,e). Provincially, a wide disparity exists and five groups can be clarified based on their stocks. A high level of stocks is found in Guangdong, Jiangsu, Shandong, Henan, Zhejiang, and Sichuan provinces (Detailed results are available in Table S3 of Supplementary Information). Material stocks in each province all show an increasing pattern during 1978–2018 (Fig. 3) (Detailed results are available in Table S4 of Supplementary Information). Further understanding of flows in the aforementioned five regions is given in Figs. 4 and 5.

With the increasing stocks, the material demand and scrap grow rapidly (Fig. 4) (Detailed results are available in Tables S5–S6 of Supplementary Information). Nationally, material demand reaches ~7.0 Gt/year, and material scrap gets to ~0.7 Gt/year in 2018. Provincially, eastern coastal provinces generate higher material demand and scrap than inland provinces. For example, Guangdong has the highest material demand and scrap of ~0.9 Gt/year and ~40.8 Mt/year in 2018. In comparison, Tibet’s material demand and scrap are only 10.8 Mt/year and 1.2 Mt/year in 2018, respectively. The material scrap is further broken down into five end-use sectors for each region. Material scrap in buildings will reach 1.1 Gt/year in 2018, accounting for ~95% of the national total scrap (Fig. 5) (Detailed results in provinces and end-use sectors are available in Table S7 of Supplementary Information).

### Comparison with existing estimates

Our results, estimated with the bottom-up accounting method, can complement the previous estimates based upon the top-down accounting method and contribute important knowledge to sustainable management of bulk materials at an unprecedented level of accuracy and resolution. We compare our stocks estimate with previous case studies on the national and provincial scales (Table 3). We find that our results are close to that of previous studies. Specifically, our results are approximately 30% lower than the top-down estimations^4,21,37^. The difference can be largely explained by the fact that the bottom-up accounting method cannot capture all the products in use in society. Data limitations, especially at the provincial level, prevent us from collecting data for commercial buildings, high-speed trains, water and environmental infrastructures, machinery possessed by small businesses, and appliances for commercial use, which may incorporate large amounts of materials. In addition, our results are generally higher than previous bottom-up accounting estimations. The amount of identified products in different end-use sectors is the main reason for the differences. For example, previous studies only identified less than 50 products in buildings and infrastructure in their studies^20,32^, while we considered 103 products in five end-use sectors in evaluation.

**Table 3:** Comparisons of stocks estimation between our results with previous estimates.

Region	Method	Year	Material	Earlier estimates	This study	Difference
China	Top-down	2010	Copper	~67.0 Mt^39^	58.5 Mt	−12.7%
China	Top-down	2010	Copper	~60 Mt^40,41^	58.5 Mt	−2.5%
China	Top-down	2010	Material stocks^a^	181.3 Gt^4^	137.3 Gt	−27.6%
China	Top-down	2010	Steel	4.1 Gt^31^	3.8 Gt	−7.3%
China	Top-down	2013	Cement	21.5 Gt^21^	19.3 Gt	−10.2%
China	Top-down	2016	Aluminium	209.5 Mt^37,42^	197.6 Mt	−5.7%
China	Bottom-up	2008	Sand^b^	28.9 Gt^20^	31.5 Gt^b^	8.2%
China	Bottom-up	2010	Steel	3.2 Gt^43^	3.8 Gt	15.7%
Shanghai	Bottom-up	2010	Material stocks^c^	561 Mt^44^	582.0 Mt^c^	3.6%
Beijing	Bottom-up	2016	Steel + Copper+Aluminium^d^	59.4 Mt^32^	60.5 Mt	2.0%

^a^Materials stocks in buildings without considering glass, rubber, and cement.

^b^Sand stocks in residential buildings and infrastructure.

^c^Material stocks in residential buildings and infrastructure.

^d^Stocks of steel, copper, and aluminium in urban area of Beijing.

From the aspect of the format, the existing stocks estimated only present the total material stocks of the whole country, or stocks of an individual material (e.g., steel, cement, or copper). Our datasets provide the products-based stocks and flows of five end-use sectors and 13 material stocks to give detailed demonstrations of China’s material use and disposal of the statue as well as its 31 provinces. Our datasets can be a more detailed supplement to the existing stocks and flows estimates.

### Limitations and future work

Unavoidably, the datasets bear uncertainties from data selection, handling, and operation.

- The first uncertainty is the material intensity. For each product, we only estimate the average material intensity for a special period. However, the material intensity appears to vary by a wide range in most product categories^32^. The great variations of material intensity contained in technological products of different sizes, weights, and functions should be considered in future work.
- The second uncertainty is the lifetime of products. Given that lifetime manifests itself in lifestyle, its impact is significant in determining the material flows^15,38^. Due to the lack of validated estimation on lifetime changes, especially at the provincial level, our study did not consider the lifetime changes. Future work can focus on regional differences in lifetime of products to reduce uncertainties in estimation.

- The third uncertainty is the assumptions when we estimate the material stocks in non-residential buildings and industrial machinery. Although these assumptions are made based on underlying reliable official data and academic research, there is a necessity to increase the results accuracy by cross-checking or enriching the input datasets in our future work.

Our future work will also focus on continuously updating the PMSFD by collecting newly published input data (the number of 103 products in five end-use sectors) based on the constructed data structures and PMSF model. Meanwhile, based on the existing input data datasets, we will estimate the stocks and flows for more materials (e.g., strategic and minor metals) to expand the PMSFD in the future to provide robust support for circular economy and sustainable development.

## Usage Notes

Since the dataset format is clear and easy to be understood, the continuous 41-year material stocks and flows records can be used to tracking and analyzing the metabolism of different materials on the provincial and national scale. The dataset can be used to assess the material efficiency/productivity and criticality, and evaluate the environmental impact in conjunction with information of production and recycling and life cycle analyses model.

The data files are documented as xlsx files, which can be readily read and processed by many software, such as Matlab, R, and Python. The 13 materials’ stocks can be distinguished into four types based on their properties, including biomass (wood), fossil materials (plastics and asphalt), metals (steel, aluminium, and copper), and non-metallic minerals (gravel, bricks, sand, cement, lime, glass, and rubber). The stocks of biomass, fossil materials, and non-metallic minerals concentrate on buildings, highways, passenger cars, and trucks. Due to the data dispersion, the ARIMA (Autoregressive Integrated Moving Average) methodology is recommended to be used when analyzing the dynamic evolution of material inflows and outflows. Furthermore, the dataset users can evaluate the stocks of other materials or the stocks of the same material in more accounting end-use sectors according to the calculation models of this study.

## Supplementary information

Supplementary Information

## Data Availability

The original input data, the amount of products quantified by per-household, per-capita, and absolute in each province, is stored as xlsx files and shared in Figshare (Input data.xlsx)^36^. Data processing is performed using MATLAB software (MatlabR2019), and the codes for creating provincial and national stocks and flows datasets are also stored in Figshare (code_calculation.m)^36^. We share these datasets and scripts for data transparency and computational reproducibility, and to assist users for further exploration and development.

## Online-only Table

**Online-only Table 1:** Sectoral and products details.

End-use sectors	Products	Products
Buildings	Urban residential buildings	Rural residential buildings
	Urban non-residential buildings	Rural non-residential buildings
Infrastructure	Expressways	≥ 500 kv voltage electricity transmission
	Highways, Class I	330 kv voltage electricity transmission
	Highways, Class II	220 kv voltage electricity transmission
	Highways, Class III	110 kv voltage electricity transmission
	Highways, Class V	35 kv voltage electricity transmission
	Hydropower electricity stations	≥ 500 kv voltage electricity transformation
	Thermal power stations	330 kv voltage electricity transformation
	Wind power stations	220 kv voltage electricity transformation
	Highway bridges	110 kv voltage electricity transformation
	Highway Tunnels	35 kv voltage electricity transformation
	Urban bridges	Urban, cable TV users
	Water supply pipelines	Rural, cable TV users
	Gas supply pipelines	Urban, landline users
	Natural gas pipelines	Rural, landline users
	Liquefied petroleum gas pipelines	Water ports
	Steam pipelines	Street lamps
	Hot-water pipelines	Urban roads
	Sewerage pipelines	Cables
Transportation equipment	Passenger cars, large	Trucks, heavy
	Passenger cars, medium	Trucks, medium
	Passenger cars, small	Trucks, light
	Passenger cars, micro	Trucks, micro
	Motorcycles	Electric locomotives
	Trailers	Diesel locomotives
	Other vehicles	Rails
	Motor vessels	Barges
Machinery	Large & medium tractors	Electromotor
	Small tractors	Fishing machine
	Large & medium towing farm machinery	Drainage & irrigation machinery
	Small tractor towing farm machinery	Harvesters
	Threshing machine	Transport power machinery
	Fishing power boats	Pumps
	Diesel engine	Agricultural power
	Industrial energy	
Domestic appliances	Urban/Rural, refrigerators	Urban, pianos
	Urban/Rural, air conditioners	Urban, other musical instruments
	Urban/Rural, washing machines	Urban, combined sound
	Urban/Rural, microwave ovens	Urban, digital video
	Urban/Rural, water heaters	Urban, camera
	Urban/Rural, smoke absorbers	Urban, fitness equipment
	Urban/Rural, TV sets	Urban, landline phones
	Urban/Rural, computers	Urban, mobile phones
	Urban/Rural, bicycles	Urban, dish-washer
	Urban/Rural, sewing machines	Urban, disinfecting cabinet
	Urban/Rural, fans	
